# AMP-Activated Protein Kinase: Do We Need Activators or Inhibitors to Treat or Prevent Cancer?

**DOI:** 10.3390/ijms22010186

**Published:** 2020-12-27

**Authors:** Fiona M. Russell, David Grahame Hardie

**Affiliations:** Division of Cell Signalling & Immunology, School of Life Sciences, University of Dundee, Dow Street, Dundee, Scotland DD1 5EH, UK; f.m.m.russell@dundee.ac.uk

**Keywords:** AMP-activated protein kinase, AMPK, biguanides, CaMKK2, cancer, kinase activators, kinase inhibitors, LKB1, tumour promoters, tumour suppressors

## Abstract

AMP-activated protein kinase (AMPK) is a key regulator of cellular energy balance. In response to metabolic stress, it acts to redress energy imbalance through promotion of ATP-generating catabolic processes and inhibition of ATP-consuming processes, including cell growth and proliferation. While findings that AMPK was a downstream effector of the tumour suppressor LKB1 indicated that it might act to repress tumourigenesis, more recent evidence suggests that AMPK can either suppress or promote cancer, depending on the context. Prior to tumourigenesis AMPK may indeed restrain aberrant growth, but once a cancer has arisen, AMPK may instead support survival of the cancer cells by adjusting their rate of growth to match their energy supply, as well as promoting genome stability. The two isoforms of the AMPK catalytic subunit may have distinct functions in human cancers, with the AMPK-α1 gene often being amplified, while the AMPK-α2 gene is more often mutated. The prevalence of metabolic disorders, such as obesity and Type 2 diabetes, has led to the development of a wide range of AMPK-activating drugs. While these might be useful as preventative therapeutics in individuals predisposed to cancer, it seems more likely that AMPK inhibitors, whose development has lagged behind that of activators, would be efficacious for the treatment of pre-existing cancers.

## 1. Introduction

The AMP-activated protein kinase (AMPK) is the central component of a signaling pathway that is conserved in essentially all eukaryotes, the exceptions being a few parasites (e.g., *Plasmodium falciparum*, the causative agent of malaria) that spend most of their life cycle living inside other eukaryotic cells, in which case the host cell provides AMPK and the parasite may therefore have been able to dispense with it [1,2,3]. AMPK is activated by various stresses that act via both classical (canonical) and non-classical (non-canonical) pathways:

1. The canonical pathway is triggered by increases in the cellular ratios of AMP:ATP and/or ADP:ATP. In most eukaryotes (apart from plants) ATP is generated from ADP mainly by mitochondrial oxidative metabolism, with a smaller contribution from glycolysis (although the latter can become more important in proliferating cells such as tumour cells). The resultant high ratios of ATP:ADP generated by these catabolic pathways represent a store of energy (analogous to a fully charged battery) that can be used to drive energy-requiring cellular processes including anabolic pathways (leading to cell growth) and progress through the cell cycle (leading to cell proliferation). An increase in the ratio of ADP:ATP indicates that the energy status of the cell is becoming compromised, and the reaction catalysed by adenylate kinases (2ADP ↔ ATP + AMP) amplifies this into even larger increases in AMP:ATP ratios [4]. Binding of AMP or ADP to a crucial site on the AMPK γ subunit (where they displace ATP) then activates AMPK by a complex mechanism (discussed in Section 2) involving phosphorylation at a conserved threonine residue on the α subunit (Thr172) by the upstream kinase and tumour suppressor, LKB1.
2. A non-canonical pathway in which AMPK is activated by glucose starvation (see Section 3.1). This activation takes place on the surface of the lysosome where AMPK is regulated in a reciprocal manner with the mammalian target-of-rapamycin complex-1 (mTORC1), a key pathway that promotes cell growth [5]. Although glucose deprivation can cause increases in AMP:ATP and ADP:ATP ratios in cells that are dependent upon glycolysis, in other cells this lysosomal pathway of AMPK activation can occur without any changes in these ratios [6].
3. A non-canonical pathway involving activation, by rising intracellular Ca^2+^, of the calcium/calmodulin-dependent kinase CaMKK2 which, like LKB1, phosphorylates Thr172 (see Section 3.2). This pathway is utilized by many hormones that switch on AMPK.
4. A non-canonical pathway in which AMPK is activated by DNA-damaging treatments often used in cancer therapy, such as etoposide [7,8] (see Section 3.3). Although the mechanisms involved in this pathway are not yet completely understood, it seems possible that it may eventually have a major impact on treatment of cancer using cytotoxic, DNA-damaging agents (see Section 5.3).
5. A non-canonical pathway (recently reported) in which AMPK is activated by direct binding of long-chain fatty acyl-CoA esters (see Section 4.3).

The downstream target proteins that are phosphorylated by AMPK are increasingly well characterized, with over 60 having been validated as direct targets in a recent review [9]. In general, AMPK down-regulates proteins involved in major anabolic pathways (such as the synthesis of fatty acids, cholesterol, triglycerides, polysaccharides, RNA and proteins), while up-regulating proteins that promote the major catabolic pathways (such as glucose uptake and glycolysis, oxidative metabolism and autophagy). As well as directly opposing anabolic pathways via phosphorylation of metabolic enzymes and transcription factors involved in these processes, AMPK activation also does so indirectly by antagonizing the pro-anabolic mTORC1 pathway [5].

The spectrum of targets that are phosphorylated may depend on the subcellular location at which AMPK activation takes place. Thus, there are already indications for different targets being modified depending on whether AMPK is activated by the lysosomal pathway (#2 above) or by the canonical pathway in the cytoplasm (#1 above) [10], while acetyl-CoA carboxylase (ACC, one of the classical targets [11] that is a widely-used marker for AMPK activation) is not phosphorylated in response to the DNA damage pathway (#4 above), presumably because ACC is not present within the nucleus [7].

## 2. AMPK—Structure and Canonical Regulation by Adenine Nucleotides

**Figure 1:**
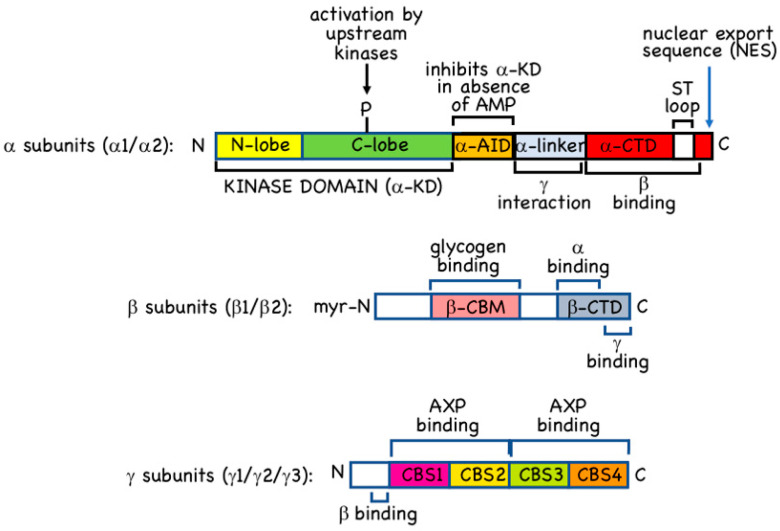
Domain maps of the three subunits of the AMPK heterotrimer: α, β and γ. Each subunit occurs as multiple isoforms. “Myr-N” refers to the myristoylated N-terminus of the β subunits. Other abbreviations/acronyms are defined in the text.

AMPK occurs universally as heterotrimeric complexes composed of catalytic α subunits and regulatory β and γ subunits (Figure 1). In humans and other vertebrates, there are two genes encoding α subunit isoforms (*PRKAA1* and *PRKAA2*, encoding α1 and α2), two genes encoding β subunit isoforms (*PRKAB1* and *PRKAB2*, encoding β1 and β2) and three genes encoding γ subunit isoforms (*PRKAG1*, *PRKAG2,* and *PRKAG3*, encoding γ1, γ2, and γ3). All combinations of α, β and γ isoforms appear to be able to form complexes [12], generating an array of 12 different heterotrimers whose diversity may be further increased by splice variants, use of alternative translation start sites, or post-translational modifications.

**Figure 2:**
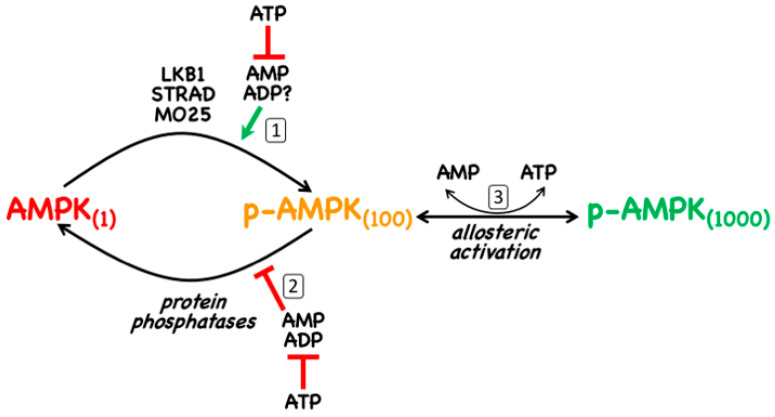
The canonical mechanism of activation of AMPK by adenine nucleotides. The action of adenine nucleotides on AMPK activity is threefold: (1) AMPK not phosphorylated on Thr172 (left) is phosphorylated by the LKB1:STRAD:MO25 complex and this reaction, which causes at least 100-fold activation, is promoted by the binding of AMP (and perhaps ADP) to AMPK itself; (2) binding of AMP or ADP to AMPK also causes a conformational change that inhibits dephosphorylation of Thr172 by protein phosphatases; (3) binding of AMP (but not ADP) causes up to 10-fold allosteric activation of AMPK. All three effects of AMP and/or ADP are antagonized by the binding of ATP. The figures in parentheses next to each form of AMPK are an indication of their approximate relative kinase activity.

As discussed in Section 1, AMPK is normally only significantly active when phosphorylated at a conserved threonine residue in the catalytic α subunit that is usually referred to as Thr172 (this numbering is derived from its position in the rat α2 subunit where it was first identified [13], although the exact numbering may vary in different species and isoforms). The canonical pathway of activation involves three distinct mechanisms, all of which are promoted by binding of AMP to the γ subunit (Figure 2): (i) promotion of Thr172 phosphorylation by the upstream kinase, LKB1; (ii) inhibition of Thr172 dephosphorylation by protein phosphatases; (iii) allosteric activation of AMPK already phosphorylated on Thr172 (note that with γ3 complexes this last effect is very small [14]). Of these three mechanisms, (ii) is mimicked by binding of ADP [15], although with γ1 and γ3 complexes only at 10-fold higher concentrations than AMP (with γ2 complexes, the effects of AMP and ADP are similar) [14]. There is general agreement that mechanism (iii) is only caused by binding of AMP, but with respect to mechanism (i) the situation is less clear. Although it has been reported that the binding of AMP or ADP promotes Thr172 phosphorylation [16,17], this may only occur significantly with γ1 complexes and then, for ADP, only at concentrations likely to be above the normal physiological range [14].

The canonical mechanism requires the presence of an upstream kinase that is constitutively active to provide a constant potential for phosphorylation of Thr172 that is, however, promoted by binding of AMP to the substrate, AMPK. In 2003–2004, three groups reported [18,19,20] that this upstream kinase was LKB1, a kinase that occurs as heterotrimeric complexes with STRAD and MO25 (both of which occur as α and -β isoforms) [18]. STRAD-α or -β, without which LKB1 is completely inactive, is a “pseudokinase” (containing a domain related to a protein kinase domain but which has no catalytic activity), while MO25, whose binding appears to stabilize the LKB1:STRAD complex, is a scaffold protein containing helical repeats related to those of Armadillo repeat proteins [21]. The finding that LKB1 was an upstream kinase for AMPK was exciting, because LKB1 was known from genetic studies to be a tumour suppressor [22], and this introduced the first clear link between AMPK and cancer, which is addressed further in Section 5 below. Consistent with LKB1 being a tumour suppressor, many human cancer cell lines carry loss-of-function mutations in the *STK11* gene that encodes it. This includes HeLa cells, the first human cells ever to be established in culture, which were derived from a case of cervical cancer in which analysis of the original tumour biopsies revealed a large deletion in the *STK11* gene [23]. Indeed, studies with HeLa cells show that agents that usually activate AMPK via the canonical mechanism fail to do so unless LKB1 is re-introduced, due to the lack of an upstream kinase providing a constant phosphorylation of Thr172 [18,19,20].

Although several candidates have been proposed (e.g., [24,25,26]), there is no general consensus as to the identity of the protein phosphatase(s) that dephosphorylate Thr172. In any case, since the effect of AMP on Thr172 dephosphorylation is due to its binding to the substrate (i.e., AMPK) rather than to the enzyme (i.e., the protein phosphatase) [24], the identity of the latter may not be critical.

We will now briefly discuss the functions of the individual subunits and domains of AMPK shown in Figure 1.

### 2.1. α Subunits

The α subunits (α1 or α2) are the catalytic subunits with kinase domains (α-KD) at their N-termini. These are typical Ser/Thr-specific protein kinase domains with small N-terminal lobes (N-lobes) and larger C-terminal lobes (C-lobes), with the binding site for the substrate Mg.ATP^2−^ in the cleft between them. Thr172 is located in the “activation loop” of the C-lobe, a region where many protein kinases must be phosphorylated to be active, and in which phosphorylation causes a conformational change that creates the docking site for the protein substrate, orienting its phosphoacceptor residue in line with the γ-phosphate of the Mg.ATP^2−^ bound in the catalytic site [27]. The α-KD is followed (Figure 1) by: (i) the autoinhibitory domain (α-AID); (ii) the α-linker, a flexible linker in an extended conformation; and (iii) a globular C-terminal domain (α-CTD). The α-AID, which contains a small bundle of three α-helices, is so-called because constructs containing just an α-KD and an α-AID are around 10-fold less active than those containing an α-KD alone. In the former constructs, the α-AID binds to both the N- and C-lobes of the α-KD, holding the latter in a less active conformation [28,29]. In heterotrimers crystallized in active conformations, the α-linker binds to one surface of the γ subunit where it contacts the activating ligand, AMP, bound in the crucial CBS3 site (see Section 2.3 below) [30]. This anchoring of the α-linker to the γ subunit by AMP requires a rotation of the α-AID away from its inhibitory site behind the kinase domain, thus explaining allosteric activation by AMP.

In the active forms of the AMPK heterotrimer that have been crystallized, the phosphorylated Thr172 residue is partly buried in a cleft between the α-KD and the α- and β-CTDs, where it is likely to be sterically protected against dephosphorylation. The conformational change that occurs when ATP replaces AMP at CBS3 (although not well understood) appears to make Thr172 more exposed to protein phosphatases, thus explaining how AMP binding protects Thr172 against dephosphorylation. How AMP binding promotes phosphorylation of Thr172 by LKB1 is less clear.

The C-terminal domains of the α subunits from vertebrates (and *Caenorhabditis elegans*) contain, just prior to their final α-helix, a serine/threonine-rich loop of around 50 residues that is not present in the α subunits from fungi, plants and insects. This region, which has been termed the “ST loop” (Figure 1), appears to be phosphorylated at multiple sites by multiple kinases [31,32,33,34,35], and our group have proposed that it represents a regulatory sequence that has been inserted during evolution of the animal kingdom [3,35]. This loop is not resolved in any of the crystal structures of AMPK heterotrimers, either because it has been deleted from the constructs used, or because it was mobile within the crystals. However, our group have provided evidence that when AMPK-α1 is phosphorylated at Ser-487 within the ST loop by the protein kinase AKT, the loop interacts with the small lobe of the kinase domain to block phosphorylation of Thr172 [35]. This provides a mechanism by which the anabolic hormones insulin and IGF-1, which activate AKT, can oppose activation of pro-catabolic and anti-anabolic signalling by AMPK [31,35].

Following the ST loop, the final α-helix of the α-CTDs from both α1 and α2 contain well-defined nuclear export sequences (NES) (Figure 1) [36]. There is good evidence that complexes containing the α2 isoform are enriched in the nuclei of several cell types [37,38,39,40], although it is also clear that α1-containing complexes can be present in the nucleus [7]. However, the presumed nuclear localization sequences (NLS) that promote nuclear entry of α1- and α2-containing complexes remain to be fully identified. The α2 isoform does contain a cluster of basic residues in the C-lobe of the α-KD (only partially conserved in α1) that was claimed to function as an NLS [41], but these results were not confirmed by others [36].

### 2.2. β Subunits

The β subunits of AMPK (β1 or β2) contain consensus sequences for myristoylation (the covalent attachment of a C14 saturated fatty acid) at their N-termini, and the human β1 and β2 subunits are indeed both modified in this manner [17,42]. N-myristoylation is thought to be required for attachment of AMPK to membranes [17,42], particularly to the cytoplasmic surface of the lysosome [6]. The exact proportion of AMPK that is bound to membranes, and whether there is a “myristoyl switch” mechanism that modulates this attachment, as with other myristoylated proteins [43], remains unclear at present. The remainder of the N-terminal regions of the β1 and β2 subunits are less well conserved and their functions unknown, but there are conserved domains in the centre and at the C-termini (Figure 1). The central domain is a carbohydrate-binding module (β-CBM) similar to those found in enzymes that metabolize starch, glycogen or related polysaccharides [44]. The β-CBM causes a proportion of AMPK within the cell to bind to glycogen particles [45,46], and the glycogen-binding site, containing two conserved tryptophan residues, is well-defined [47]. Glycogen binding would cause co-localization of AMPK with glycogen-bound substrates such as: (i) glycogen synthases [48,49], key enzymes of glycogen synthesis that are inactivated by AMPK; (ii) R5/PTG/PPP1R3C [50], a protein phosphatase-1 regulatory subunit that targets the catalytic subunits to glycogen; and (iii) genethonin-1/STBD1 [51], which is involved in glycogen degradation by the lysosome. Surprisingly, knock-in mutations in mice that abolished glycogen binding in vivo, by mutation of key tryptophan residues in AMPK-β1 or -β2, did not reveal any significant changes in glycogen content in liver or muscle, although other metabolic defects were observed [52]. Notably, the β1 W100A mice showed increased fat content in liver (where β1 is the predominant isoform), while the β2 W98A mice showed increased fat content in skeletal muscle (where β2 is the predominant isoform) as well as increased overall fat mass. These changes were associated with reduced expression of AMPK-α and -β1 in the liver of β1 W100A mice, and of AMPK-α and -β2 in the muscle of β2 W98A mice; both effects were associated with reduced total AMPK activity. The authors suggested that the binding of AMPK to glycogen provides a reserve pool of the kinase more resistant to degradation, that would potentially become available to phosphorylate non-glycogen bound targets when glycogen stores became degraded [52]. If this interesting proposal is correct, AMPK can be regarded as a type of “glycogen-sensor”.

### 2.3. γ Subunits

The γ2 and γ3 isoforms contain lengthy N-terminal regions that are unrelated to each other and are not present in the γ1 isoform; although their exact functions are not known, they may be involved in targeting the complexes to specific subcellular locations, particularly in striated muscles that express high levels of either γ2 (cardiac muscle) or γ3 (skeletal muscle). All three γ subunits also contain four tandem repeats (Figure 1) of a sequence of around 60 residues termed a CBS repeat [53]. CBS repeats occur in about 50 proteins encoded in the human genome, and also occur in bacteria and archaea [54]. They almost invariably occur as tandem repeats, and the two repeats of single pairs have been shown to associate together to form pseudodimers, with two pseudosymmetrical binding sites for potential ligands in the intervening cleft [54]. The ligands known to bind there usually contain adenosine, and include ATP, AMP and S-adenosyl methionine [54,55]. The AMPK-γ subunits are unusual in having four repeats, labelled CBS1 through CBS4 (Figure 1). The two pseudodimers formed by CBS1:CBS2 and CBS3:CBS4 come together head-to-head to form a disc-shaped structure with one repeat in each quadrant, containing four potential nucleotide-binding sites lining a narrow aqueous channel through the centre. These binding sites are now numbered [56] according to the number of the CBS repeat that binds the adenosine moiety of the bound nucleotide (the phosphate group(s) can bind to side chains from other repeats). Of these, the CBS1 site may be permanently occupied by ATP [57], the CBS2 site appears to be always unused, while the CBS4 site (confusingly, originally called site 3 [58]) appears to be permanently occupied by AMP [58]. This leaves CBS3 as the critical site which, when occupied by AMP in the active form of the kinase, contacts the α-linker (Section 2.1). Displacement of AMP by ATP at the CBS3 site is thought to convert the kinase to a less active conformation that is also more susceptible to Thr172 dephosphorylation. The functions of the CBS1 and CBS4 sites are less clear, although it has been proposed that their permanent occupancy by ATP and AMP (respectively) may alter the conformation of the CBS3 site such as to increase its affinity for AMP relative to ADP or ATP [57]. This helps to explain how AMPK achieves the difficult task of sensing changes in AMP in the presence of much higher concentrations of ADP and ATP. An additional factor in this discrimination between nucleotides is that the CBS sites preferentially bind free ATP rather than its complex with Mg^2+^, in which some of the favourable interactions between the negatively-charged phosphate groups of ATP and the positively-charged basic side chains of the CBS sites would be sterically blocked by the Mg^2+^ ion; note that only a small proportion of cellular ATP (around 10%) normally occurs as the Mg^2+^-free form.

## 3. Non-Canonical Regulation of AMPK

### 3.1. Non-Canonical Regulation by Glucose Starvation

**Figure 3:**
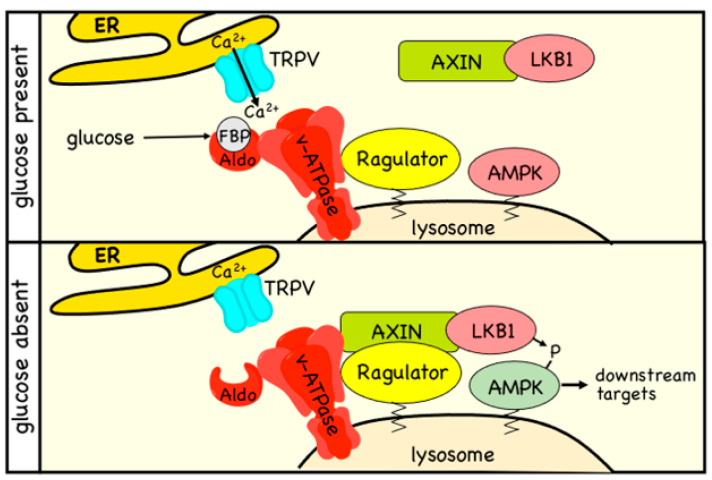
Non-canonical mechanism of activation of AMPK by glucose starvation. The model is based on that in Li et al. [60]. In the presence of high glucose (top panel), a large proportion of aldolase (Aldo), which is bound to the v-ATPase proton pump on the lysosome, will have its substrate, the glycolytic intermediate fructose-1,6-bisphosphatase (FBP), bound to it. This causes the opening of TRPV Ca^2+^ channels located at ER:lysosome contact sites, maintaining the function of the v-ATPase and promoting mTORC1 activity via the Ragulator complex, which is anchored on the surface of the lysosome via lipid modifications. When glucose is absent or limiting (lower panel), aldolase will not be fully occupied by FBP, and it interacts with and inhibits the neighbouring TRPV channels. The v-ATPase is now inhibited, allowing an interaction between the Ragulator and the cytoplasmic Axin:LKB1 complex, and bringing LKB1 into the proximity of AMPK, which is then phosphorylated and activated. In this version of the model, a pool of AMPK is viewed as being permanently located at the lysosome due to the N-terminal myristoylation of the β subunit.

Since it was first demonstrated in 1998 [59], glucose starvation of mammalian cells has been widely used as a treatment to activate AMPK. Most researchers seem to have assumed that the effect is caused by a reduced input of glucose into glycolysis, leading to ATP depletion and consequent activation by the canonical pathway. While this can indeed occur in some cell types that are dependent on glycolysis for ATP production, in other cells (e.g., mouse embryo fibroblasts) removal of glucose from the medium still activates AMPK without any associated increases in AMP:ATP or ADP:ATP ratios, as long as alternative carbon sources such as glutamine and pyruvate are present in the medium [6]. Even in those cells where there are increases in AMP:ATP and ADP:ATP ratios triggered by glucose starvation (e.g., HEK-293 cells), AMP-insensitive mutants of AMPK (which carry a mutation in the crucial CBS3 site on the γ subunit) can still be activated by the treatment [6]. Thus, it is clear that, in many cells, there is an adenine nucleotide-independent component of the mechanism by which AMPK is activated during glucose starvation. In such cases, activation is thought to occur via a complex mechanism (Figure 3) involving the direct sensing of the glycolytic intermediate fructose-1,6-bisphosphate (FBP) by FBP aldolase, and the recruitment of AMPK to a “super-complex” on the lysosomal membrane involving the vacuolar-ATPase, the Ragulator complex, Axin, LKB1, and AMPK [3].

Sensing of carbohydrate availability may have been one of the ancestral roles of the AMPK system, since the orthologue in budding yeast is potently activated by glucose removal from the medium [61,62]. Similarly, the orthologues in green plants are required for responses to darkness [63,64], which blocks the supply of carbohydrate from the Calvin cycle of photosynthesis. Nevertheless, the non-canonical pathway for glucose sensing cannot be completely conserved between mammals and yeast, because there appear to be no orthologues of Axin in the latter. In addition, neither the yeast [62] nor the plant [65] orthologues of AMPK are allosterically activated by AMP, although there is evidence that their dephosphorylation at the residue equivalent to Thr172 is inhibited by binding of ADP [66] or AMP [67], respectively. Thus, even the canonical pathway is not completely conserved across all eukaryotes.

### 3.2. Non-Canonical Regulation by CaMKK2

**Figure 4:**
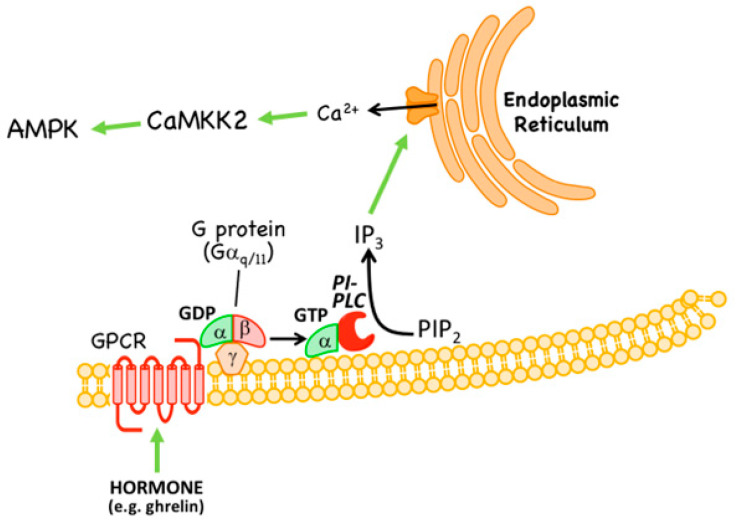
Non-canonical mechanism of activation of AMPK by hormones that trigger intracellular Ca^2+^ release. Hormones such as ghrelin bind to a G protein-coupled receptor (GPCR) that is coupled via G proteins containing Gα_q/11_ to activation of phosphatidylinositol-specific phospholipase C (PI-PLC). This triggers release of inositol-1,4,5-trisphosphate (IP_3_) from the plasma membrane phospholipid phosphatidylinositol-4,5-bisphosphate (PIP_2_). IP_3_ diffuses to the endoplasmic reticulum where it binds to IP_3_ receptors, triggering release of Ca^2+^ ions. These in turn activate Ca^2+^/calmodulin-dependent kinase kinase-2 (CaMKK2) which phosphorylates AMPK at Thr172, causing its activation.

Although the basal phosphorylation of Thr172 is very low in HeLa cells (which lack LKB1), it is not eliminated completely and can be promoted by agents that increase intracellular Ca^2+^ [68]. This suggested the existence of an alternative upstream kinase to LKB1 in these cells that was activated by Ca^2+^. This was soon traced to phosphorylation of Thr172 by the Ca^2+^/calmodulin-dependent kinase, CaMKK2 [68,69,70], which had originally been identified as an upstream kinase for another Ca^2+^/calmodulin-dependent kinase, CaMKI [71]. The Ca^2+^ → CaMKK2 → AMPK pathway occurs, for example, as a response to hormones and agonists sensed by G protein-coupled receptors and other receptor types that are coupled to release of inositol-1,4,5-trisphosphate (IP_3_) from the plasma membrane, which in turn triggers release of Ca^2+^ from the endoplasmic reticulum (Figure 4). Such agonists include: in endothelial cells, thrombin acting at protease-activated receptors and vascular endothelial cell growth factor acting at VEGF receptors [72,73]; and, in specific neurons of the hypothalamus, the “hunger hormone” ghrelin acting at GHSR1 receptors [74]. The last effect is important in promotion of appetite during fasting [75]. It is intriguing that a signalling pathway that may have evolved to sense carbohydrate deficiency at the single cell level (see Section 3.1) should have become adapted to fulfill a similar role at the whole-body level in mammals, where feeding is now a complex behavior involving multiple hormonal stimuli.

### 3.3. Non-Canonical Regulation by DNA Damage

**Figure 5:**
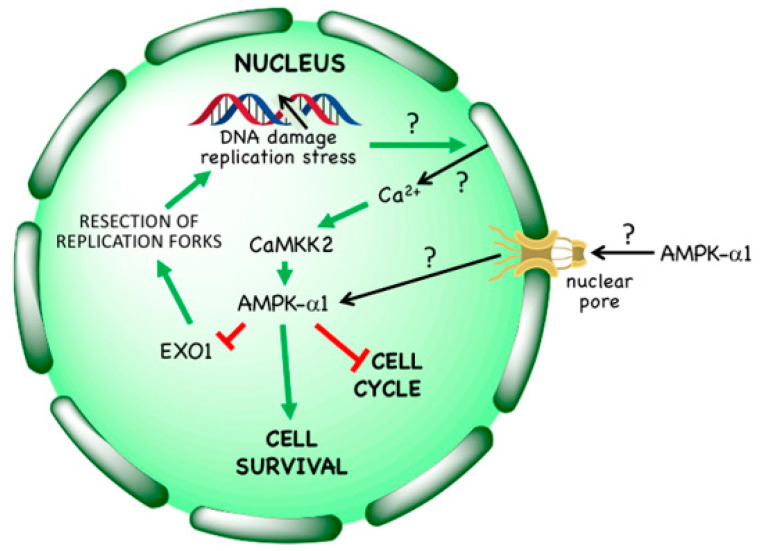
Non-canonical mechanism of activation of AMPK in the cell nucleus by treatments that cause DNA damage. Agents such as ionizing radiation, cytotoxic drugs or DNA synthesis inhibitors cause DNA damage, either directly or following replicative stress. This causes release of Ca^2+^ ions within the nucleus, which activates CaMKK2 within that compartment. AMPK complexes containing AMPK-α1 also appear to translocate from the cytoplasm to the nucleus, where they are activated by CAMKK2-dependent phosphorylation at Thr172. AMPK activation enhances cell survival; it causes a G1 phase cell cycle arrest, and triggers phosphorylation of EXO1, limiting its ability to cause excessive resection at stalled replication forks. As indicated by question marks, several aspects of this mechanism remain unresolved: (i) how is the DNA damage sensed? (ii) what is the source of Ca^2+^ and its mechanism of release into the nucleus? and (iii) how is the translocation of AMPK-α1 to the nucleus achieved and regulated? The source of nuclear Ca^2+^ release is shown here as the nuclear envelope, but this is also unconfirmed.

In 2008 it was reported that AMPK was activated in response to the anti-cancer agent etoposide [76], and this was later also observed on treatment of cells with hydroxyurea, aphidicolin, bleomycin or ultraviolet light [8], as well as ionizing radiation [77]. These treatments either directly cause single or double strand breaks in DNA or are inhibitors of DNA synthesis that cause stalling of replication forks, which can in turn lead to DNA strand breaks; several of them are used as cytotoxic therapies in cancer. It was originally proposed that activation of AMPK by etoposide required the phosphatidylinositol 3-kinase–like kinase ATM [78], which is known to be activated by double strand breaks in DNA and to phosphorylate the upstream kinase for AMPK, LKB1 [79]. However, this cannot be the primary mechanism, because both etoposide [7,76] and ionizing radiation [77] still activate AMPK even in LKB1-null tumour cells such as HeLa cells, while AMPK activation by etoposide was not blocked by the ATM inhibitor KU-55933 [7]. Indeed, AMPK activation by etoposide or hydroxyurea in LKB1-null cells is mediated by Thr172 phosphorylation catalysed by CaMKK2, and is associated with increases in Ca^2+^ within the nucleus (Figure 5) [7,8]. Interestingly, only AMPK complexes containing the α1 isoform are activated by etoposide, even if α2 is also present in the cells under study [7]; the explanation for this isoform selectivity is not known at present. Note that even in cells that express LKB1 (i.e., most normal cells), LKB1 is probably not responsible for phosphorylation and activation of AMPK within the nucleus, because binding of LKB1 to STRAD and MO25, which is essential for its activity, also causes its exclusion from the nucleus [80].

Intriguingly, activation of AMPK in LKB1-null cells (using the Ca^2+^ ionophore A23187 to activate CaMKK2) provides significant protection against etoposide-induced cell death, an effect that was abolished in AMPK knockout cells [7]. Also consistent with this, AMPK knockout cells are more susceptible to cell death induced by hydroxyurea [8]. Why does AMPK activation enhance cell survival in response to DNA damage? One reason might be that AMPK activation using various treatments such as AICA riboside [81], glucose starvation [82], or activation of the Ca^2+^/CaMKK2 pathway [7] causes a G1 cell cycle arrest, thus restricting entry of cells into S phase where they would be more susceptible to DNA replication stress or DNA damage. This interpretation was supported by findings that the G1 cyclin-dependent kinase inhibitor, palbociclib, caused a very similar degree of protection against etoposide-induced cell death as AMPK activation, but only in those cell lines where it also caused a G1 arrest [7]. An alternative explanation (not necessarily mutually exclusive) came with the recent identification by Li et al. [8] of the exonuclease EXO1 as a direct nuclear target for AMPK. These authors reported that EXO1 became phosphorylated at Ser746 by AMPK (perhaps also in part by the kinase Chk1) in response to treatment with hydroxyurea or aphidicolin. This phosphorylation was reported to trigger EXO1 binding to 14-3-3 proteins, preventing its recruitment to chromatin. This would, in turn, protect against excessive resection of replication forks by EXO1, thus maintaining genome stability when DNA synthesis was inhibited. Consistent with this, in EXO1 knockout cells reconstituted with an S746A mutant and treated with the ribonucleotide reductase inhibitor hydroxyurea, there were elevated levels of fork resection and chromosomal abnormalities, including chromosome breakage and fusion, compared with wild type controls. Moreover, the increased sensitivity of AMPK knockout cells to cell death induced by hydroxyurea could be largely reversed by siRNA knockdown of EXO1 [8].

Taken together, these findings suggest that the activation of AMPK in response to DNA damage aids cell survival. A corollary of this is that AMPK inhibitors should potentiate the effects of many of the DNA damaging, cytotoxic treatments that are used in cancer therapy. This possibility is considered further in Section 5.

## 4. Pharmacological Activation and Inhibition of AMPK

The idea that activation of AMPK might be useful in treatment of disorders of whole body energy balance such as obesity and Type 2 diabetes, which appears to have been first proposed in 1999 [83], led to a drive towards the development of AMPK activators by pharmaceutical and biotechnology companies. These campaigns have now given rise to several different classes of activator that are discussed below. There has been much less emphasis on the development of inhibitors, although (as mentioned in the previous paragraph) these may now be regarded as good candidates for the treatment of some cancers. The limited current progress in this direction is discussed in Section 4.4 below.

### 4.1. AMPK Activators: Pro-Drugs That Are Converted to AMP Analogues

**Figure 6:**
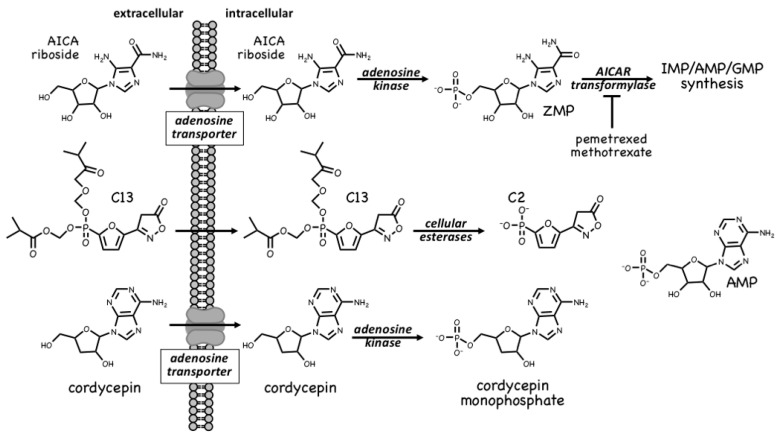
Activation of AMPK by pro-drugs that are taken up into cells and converted into AMP analogues. TOP: AICA riboside is an adenosine analogue that is taken up into cells via adenosine transporters and converted by adenosine kinase into the equivalent nucleotide, ZMP, an AMP analogue that mimics the effects of AMP on the AMPK system. MIDDLE: C2 is a phosphonate analogue of AMP that is administered in an esterified form (C13) that diffuses across the plasma membrane and is converted by cellular esterases into C2. BOTTOM: cordycepin is an adenosine analogue that is taken up into cells via adenosine transporters and converted by adenosine kinase into the equivalent nucleotide, cordycepin-5′-monophosphate, an AMP analogue that mimics effects of AMP on the AMPK system.

The first pharmacological agent shown to activate AMPK was 5-aminoimidazole-4-carboxamide ribonucleoside [84,85,86], which those in the AMPK field often refer to as AICAR. Unfortunately, this acronym can cause confusion because researchers in the field of nucleotide metabolism use the same term to refer to the monophosphorylated nucleotide, also sometimes known as ZMP; to avoid this, we will refer to the nucleoside as AICA riboside and to the nucleotide as ZMP. The incubation of cells in vitro (or injection of animals in vivo) with AICA riboside usually leads to activation of AMPK because the riboside is rapidly taken up into cells by adenosine transporters [87] and rapidly converted to ZMP by adenosine kinase [88] (Figure 6). ZMP, which is an AMP analogue, then mimics the multiple effects of AMP on the AMPK system, including allosteric activation and protection against Thr172 dephosphorylation. Although ZMP is around 50-fold less potent in these effects than AMP itself, AICA riboside is nevertheless effective at activating AMPK in many cells because ZMP accumulates to millimolar concentrations in their cytoplasm [86]. AICA riboside was historically used as a means of artificial activation of AMPK in vitro and in vivo, and is still sometimes used today. However, it is perhaps not surprising that millimolar levels of any ligand can have off-target effects. For example, ZMP mimics the effects of AMP on other AMP-sensitive enzymes, including the glycogenolytic enzyme glycogen phosphorylase in cardiac muscle [89] and the gluconeogenic enzyme fructose-1,6-bisphosphatase in the liver [90]. Indeed, some of the effects of AICA riboside on hepatic glucose production, which were originally ascribed to AMPK activation, can now be put down to inhibition of fructose-1,6-bisphosphatase by ZMP [91,92].

ZMP is, in fact, a naturally-occurring metabolite in the pathway of *de novo* purine nucleotide synthesis, being converted in two further steps to IMP, the common precursor for AMP and GMP (Figure 6). These two steps are catalysed by the enzymes AICAR transformylase and IMP cyclohydrolase, which are carried on a single polypeptide chain encoded by the *ATIC* gene. The rapid metabolism of ZMP to IMP explains why AMPK is not activated by AICA riboside in some cell types, especially in proliferating cells that have a high capacity for de novo nucleotide biosynthesis. AICAR transformylase (note that AICAR here refers to the nucleotide, i.e., ZMP) uses N^10^-formyltetrahydrofolate to add an aldehyde group to what is then converted by IMP cyclohydrolase into the 6-membered ring of IMP. Because of this, AICAR transformylase is inhibited by folate analogues such as methotrexate and pemetrexed, which are used in the treatment of some cancers and autoinflammatory disorders. While the primary target of these antifolate drugs is thought to be thymidylate synthase and hence DNA synthesis, AICAR transformylase may be a secondary target. Thus, pemetrexed has been shown to activate AMPK in leukaemia cells due to inhibition of AICAR transformylase and consequent accumulation of its substrate ZMP [93], while in HEK-293 cells, where AICA riboside has little effect on its own because of rapid metabolism of ZMP by AICAR transformylase, the ribonucleoside does activate AMPK dramatically in the presence of the AICAR transformylase inhibitor, methotrexate [94]. Whether these effects on AMPK explain any of the anti-cancer actions of antifolate drugs remains unclear at present.

A much more selective AMPK activator that works through a different pro-drug mechanism is C13, which contains a phosphonate group esterified on two of its oxygen atoms (Figure 6). This modification makes C13 cell-permeable but, once inside cells, it is converted by intracellular esterases into C2, a phosphonate analogue of AMP that, remarkably, is at least two orders of magnitude more potent as an allosteric activator of AMPK than AMP itself [95]. An explanation for this surprising finding is that although its binding site on the AMPK-γ subunit overlaps with that of AMP, the two nucleotides bind in different orientations [96]. Another notable feature of C2 is that it is almost completely selective for AMPK complexes containing the α1 isoform, with little or no effect on α2 complexes. One major advantage of the use of C13, compared with AICA riboside, is that its active metabolite C2 does not affect the regulation of other AMP-sensitive enzymes such as glycogen phosphorylase and fructose-1,6-biphosphatase [97].

A final compound that activates AMPK by a pro-drug mechanism related to that of AICA riboside is cordycepin, also known as 3′-deoxyadenosine (Figure 6). Cordycepin is an adenosine analog derived from species of the genus *Cordyceps*, which are parasitic fungi (highly prized in traditional Chinese medicine) that infect insect larvae such as caterpillars and (rather gruesomely!) consume them from within [98]. Cordycepin is an adenosine analogue that is taken up into cells and converted by cellular metabolism into mono-, di- and tri-phosphates. Since it lacks a hydroxyl group at the 3′-position on the ribose ring, if cordycepin is incorporated into RNA it will cause chain termination. Poly(A) polymerases, which add the poly(A) tails to mRNA, seem to accept cordycepin in place of adenosine particularly readily, so that treatment of cells with cordycepin reduces the lengths of the poly(A) tails, and hence the stability, of many mRNAs [99]. This may be the main reason why cordycepin has cytotoxic effects to reduce cell viability. However, two groups reported in 2010 that cordycepin treatment also activated AMPK [99,100]. Our group has provided evidence that this occurs because cordycepin is taken up into cells via adenosine transporters, and is converted by adenosine kinase into cordycepin monophosphate (Figure 6), which then mimics the multiple effects of AMP to activate AMPK [88]. Cells lacking AMPK are also more sensitive to the cytotoxic effects of cordycepin, suggesting that the ability of the compound to activate AMPK normally ameliorates its cytotoxic effects.

### 4.2. AMPK Activators: Indirect Activation via Inhibition of ATP Synthesis

**Figure 7:**
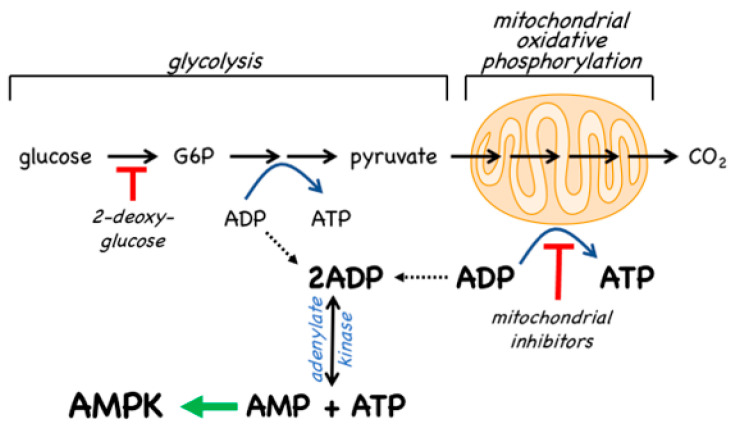
Indirect activation of AMPK by drugs that interfere with cellular ATP synthesis. Depending on the cell type, drugs that inhibit glycolysis (e.g., 2-deoxyglucose) or that inhibit mitochondrial oxidative phosphorylation (especially Complex I inhibitors such as metformin, phenformin and berberine) cause an accumulation of ADP relative to ATP. This in turn causes displacement of the adenylate kinase reaction towards AMP and ATP, increasing AMP dramatically while having much smaller effects on ADP and ATP levels. The increase in AMP (and perhaps also ADP) relative to ATP then activates AMPK by the canonical mechanism shown in Figure 2.

Since the inhibition of ATP synthesis will cause increases in the ADP:ATP ratio that are amplified by adenylate kinases into even larger increases in the AMP:ATP ratio, this will inevitably also cause a secondary, indirect activation of AMPK (Figure 7). For example, in cells that are partly reliant on glycolysis for ATP production, addition of the glycolytic inhibitor 2-deoxyglucose causes rapid AMPK activation [101]. However, the major generator of ATP in most animal cells is not glycolysis but mitochondrial oxidative metabolism, and many pharmacological agents that activate AMPK do so indirectly by inhibiting the latter (Figure 7). In particular, Complex I of the respiratory chain (NADH–ubiquinone oxidoreductase) is a remarkable membrane-bound multiprotein machine with 14 core subunits and around 30 accessory subunits [102]. It is perhaps not surprising that many xenobiotic compounds that are hydrophobic in nature should find binding sites within this complex, where they cause its inhibition, with consequent downstream activation of AMPK. Many compounds known to activate AMPK by this mechanism are secondary products of plants and may be produced by the plant to poison herbivorous insects and other animals, and thus deter grazing. These compounds are often stored in the cell wall or vacuole of the producing plant cell, where they will not come into contact with the mitochondria of the plant itself [103]. An example of a natural plant product that activates AMPK is galegine [104], which is derived from the plant *Galega officinalis*. The latter (also known as Goat’s Rue, presumably because it is poisonous to goats!) was recommended as a herbal remedy in a medical text [105] published as long ago as 1640 by John Parkinson, the court physician to King James I of England. Galegine itself, an isoprenyl guanidine, turned out to be too toxic for human use, but in the 20th century various biguanide derivatives, including metformin and phenformin, were synthesized. In the second half of the century these became mainstays of the treatment of Type 2 diabetes, although phenformin was withdrawn in the late 1970s because in rare cases it was associated with a life-threatening lactic acidosis. In 2000, two groups reported that metformin and phenformin were inhibitors of Complex I of the respiratory chain [106,107] (providing, incidentally, an explanation for the lactic acidosis associated with phenformin use), and the following year it was reported that metformin activated AMPK [108]. Although there have been various proposals to explain the mechanism of action of biguanides other than by inhibition of Complex I and other than by AMPK activation [109,110], there is general agreement that these drugs do indirectly activate AMPK.

Traditional herbal medicines are a particularly rich source of natural plant products that activate AMPK—a 2016 review listed at least 100 such compounds [111], and more are still being reported in multiple papers every week. In most cases, the mechanism(s) by which these compounds activate AMPK have not been established. However, berberine [112] and arctigenin [113] have been shown to inhibit Complex I of the respiratory chain while galegine, quercetin, resveratrol, berbamine, and mangiferin have all been suggested to activate AMPK indirectly by altering cellular adenine nucleotide ratios [114,115,116].

### 4.3. AMPK Activators: Drugs and Metabolites Binding at the AdaM Site

**Figure 8:**
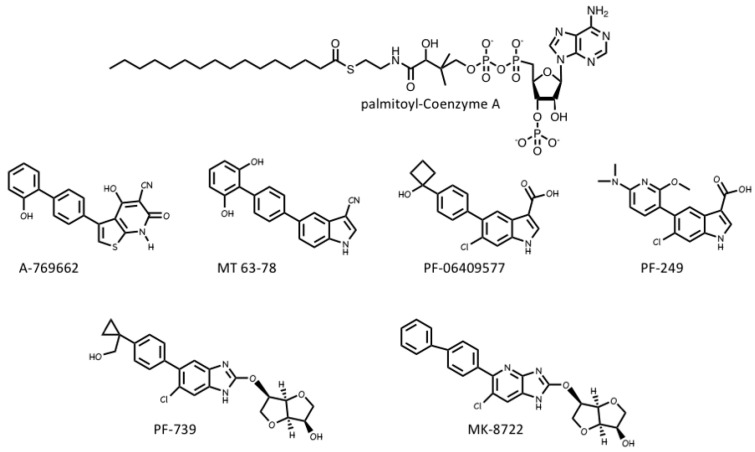
Natural and pharmacological activators of AMPK that bind in the ADaM site. TOP: palmitoyl-CoA (and other long chain saturated and monounsaturated acyl-CoA esters) are believed to the natural ligands that bind at the ADaM site, but they only activate β1-containing complexes. MIDDLE: synthetic activators that are selective for β1-containing complexes. BOTTOM: synthetic pan-β activators that activate both β1- and β2-containing complexes.

Several pharmaceutical companies have isolated direct activators of AMPK from compound libraries via high-throughput screens that searched for allosteric activators of the purified kinase (Figure 8). First-in-class was A-769662, which had favourable effects on several metabolic parameters when injected into genetically obese (ob/ob) mice, but had poor oral availability [117]. Mechanistic studies suggested that although A-769662 binding had similar effects to AMP (causing both allosteric activation and inhibition of Thr172 dephosphorylation) it bound at different site(s), and the interactions appeared to involve the β subunit because mutations in the latter abolished activation by A-769662 but not AMP [118,119]. Also supporting a role for the β subunit were findings that A-769662 only activated AMPK complexes containing β1 and not the β2 isoform [120]. Additional screens led to the development of more potent activators in this class, such as MT 63-78 [121], PF-06409577 [122], PF-249 [123], PF-739 [124], and MK-8722 [12] (Figure 8). Several of these compounds have much better oral availability than A-769662 and some, e.g., PF-739 [124], and MK-8722 [12], are termed “pan-β” activators in that they activate β2-containing complexes almost as well as β1 complexes. Because β2 is the major β subunit isoform expressed in skeletal muscle, both PF-739 and MK-8722 are effective in promoting glucose uptake by skeletal muscle, and this appears to be why they are more efficacious in ameliorating metabolic defects in animal models of obesity and Type 2 diabetes than β1-selective activators, such as A-769662 or PF-249 [12,124].

The crystallization of human AMPK complexes with 991 (a lead compound identified during development of MK-8722), or A-769662 or its halogenated analogues, finally definitively identified the binding site for these compounds [30,125]. They bind in a deep hydrophobic cleft located between the β-CBM (the opposite surface to the glycogen-binding site) and the N-lobe of the α-KD (the opposite surface to the MgATP^2-^-binding site). Because this site lies between the α and β subunits of the complex, it is unique to AMPK, and therefore a potentially specific target site for therapeutics. A curious feature of this site is that essentially all of the compounds that were initially found to activate AMPK by binding to it were derived from libraries of synthetic small molecules, rather than natural products. However, most researchers in the field believe these compounds must be mimicking a metabolite that occurs naturally in mammalian cells, hence its designation as the allosteric drug and metabolite (ADaM) site [126]. One natural product that does bind the ADaM site is salicylate, an active metabolite of the drug acetyl salicylic acid (ASA or aspirin). Salicylate, or derivatives such as salicin and methyl salicylate, are used by higher plants as hormones that signal infection by pathogens [127]. Salicylate was found to activate AMPK in a manner competitive with A-769662 [128], and an iodinated analogue of salicylate bound to the same site as A-769662 [125]. However, salicylate would not occur naturally in animals, except perhaps if plants suffering from a pathogen infection had been ingested. 

Recently, it has been suggested by Pinkosky et al. [129] that the natural ligands that activate AMPK by binding to the ADaM site may be long-chain fatty acyl-CoA esters (LCFA-CoAs). Ironically, this brings the story of regulation of AMPK full circle, as one of the first papers to define AMPK and demonstrate its sensitivity to AMP also noted that its activation by an upstream kinase (at that time unidentified) was enhanced by palmitoyl-CoA [130]. Subsequently, activation of AMPK by LCFAs was found in perfused rat heart in vitro and in rat liver in vivo [131,132], and it was also reported that CoA esters of LCFAs or LCFA analogues enhanced phosphorylation of AMPK by LKB1 [133,134]. 

In this latest paper [129], the structural basis for the regulation of AMPK by LCFAs has been addressed. It was found that micromolar concentrations of LCFA-CoAs containing saturated or mono-unsaturated fatty acids of 12 carbons or more activated AMPK, whereas the corresponding free acids or carnitine esters did not. Moreover, like A-769662, palmitoyl-CoA (C16, saturated) only activated β1-containing complexes, and β-CBM mutations that perturbed the ADaM site, but not γ subunit mutations affecting the nucleotide-binding sites, eliminated or reduced palmitoyl-CoA-induced AMPK activation. Note that, in cells subject to glucose starvation, LCFA oxidation in mitochondria would represent a crucial alternative source of ATP. The activation of AMPK by LCFA-CoAs, derived either from external sources or from breakdown of stored triglycerides, would therefore represent a type of “feed-forward” activation that would trigger enhanced LCFA oxidation via phosphorylation of acetyl-CoA carboxylases (ACC1/ACC2), with consequent relief of malonyl-CoA-mediated inhibition of LCFA uptake into mitochondria by the carnitine:palmitoyl-CoA transferase system [135]. Consistent with this, Pinkosky et al. [129] found that incubation of mouse hepatocytes with LCFAs promoted phosphorylation of ACC1/ACC2 at the equivalent AMPK sites (S79/S221), while oral administration of a triglyceride and phospholipid emulsion in vivo promoted fat oxidation in wild type mice, but not in knock-in mice in which both S79 and S221 had been mutated to alanine. If this intriguing model is correct, AMPK represents not only a sensor of cellular energy status and an indirect sensor of the availability of glucose (and perhaps also glycogen, see Section 2.2), but also a direct sensor of fatty acid availability.

### 4.4. AMPK Inhibitors

Perhaps because the suggestion that inhibitors of AMPK might be useful in the treatment of cancer has only been made fairly recently (see Section 5.3 below), there has been much less progress in their development than for AMPK activators. In 2001, a screen identified compound C as an inhibitor of AMPK that appeared to bind to the MgATP^2-^-binding site in the kinase domain [108]. It did not inhibit any of a panel of 5 other protein kinases [108] and, as a result, is still often claimed to be a “highly selective” inhibitor of AMPK. However, this is not the case; one early indication that compound C has multiple targets was that it is identical with dorsomorphin, which was identified in an independent screen for compounds that perturb dorsoventral axis formation in zebrafish and is an inhibitor of BMP (bone morphogenetic protein) signalling acting independently of AMPK [136]. Moreover, in a screen of around 70 protein kinases, Bain et al. [137] reported that at least ten were inhibited by compound C to a greater extent than AMPK, while in recent screens of more than 100 protein kinases available via the MRC Kinase Profiling Inhibitor Database [138], up to 30 were inhibited to a greater extent than AMPK. As stated by Bain et al. [137]: “the use of this compound to identify potential functions of AMPK is not recommended”. The field therefore awaits the development of a truly specific inhibitor and, despite the widespread use of compound C as an AMPK inhibitor in the literature, currently the only way to be sure that AMPK is involved in a cellular process is to use genetic approaches such as gene knockouts.

## 5. Is AMPK a Tumour Suppressor or a Tumour Promoter?

The discovery that AMPK represented a major signalling pathway downstream of LKB1 [18,19,20] immediately suggested that it might mediate some of the tumour suppressor effects of LKB1. The gene encoding LKB1 in humans (*STK11*) had been shown a few years earlier to be the primary gene mutated in Peutz–Jeghers syndrome, a hereditary predisposition to cancer [139]. Patients with this syndrome are usually heterozygous for loss-of-function mutations in *STK11* and suffer from the growth of numerous polyps in the intestine. These are classed as hamartomas and are usually benign, but frequent surgery may be required to correct them, and individuals with the syndrome also carry a greatly increased risk of malignant tumours occurring at many different locations [22]. In addition, somatic mutations in *STK11* are now known to occur quite frequently in non-inherited cancers. For example, the gene is mutated in 10–20% of all cases of human lung cancer [1,140,141,142].

It is worth noting that LKB1 is also an upstream kinase for a family of twelve protein kinases known as the AMPK-related kinases or ARKs. These are related to AMPK within their kinase domains and have critical threonine residues equivalent to Thr172 of AMPK within their highly conserved activation loop sequences [143,144]. In principle, any one of these ARKs could exert tumour suppressor effects of LKB1, and indeed there is evidence that two of them, MARK1 and MARK4, are involved in the epithelial-to-mesenchymal transition, which is important in tumour metastasis [145]. However, AMPK-α1 and -α2 are the only members of this family for which where there is clear evidence that they cause inhibition of cell growth and cell proliferation when activated, and these are characteristics expected for tumour suppressors.

### 5.1. Evidence from Mouse Models That AMPK Is a Tumour Suppressor

Strong evidence that AMPK can act as a tumour suppressor came from genetic manipulation of certain mouse models, particularly of B- and T-cell lymphomas. Analysis of these cancers has the advantage that cells of the haemopoietic lineage only express the α1 and not the α2 isoform of the AMPK catalytic subunit, so that to study the effect of lack of AMPK it is only necessary to knock out one gene (*Prkaa1*) rather than two. Initial results came from the study of B cell lymphomas induced by over-expression of the Myc oncogene from a B-cell-specific promoter [146]. Consistent with AMPK-α1 being a tumour suppressor, loss of both alleles of *Prkaa1* caused a notable acceleration in the onset of lymphomas, while loss of a single allele caused an intermediate effect. Studies with lymphoma cells over-expressing Myc in vitro also revealed that knockdown of AMPK-α1 using shRNA was associated with a hyperactivation of the mTORC1 pathway, which was to be expected since AMPK is known to restrain mTORC1 activity via multiple mechanisms [5]. This in turn led to increased glucose uptake and flux through the glycolytic pathway to provide precursors for biosynthesis—an example of the well-known Warburg effect that is often displayed by tumour cells and other rapidly proliferating cells. This was proposed to result from mTORC1 activation causing increased expression of HIF-1α (hypoxia-inducible factor-1α), a transcription factor that increases expression of glycolytic enzymes [146].

Another study involved crossing mice with knockouts of the genes encoding p53 (*Trp53*) and AMPK-β1 (*Prkab1*), the major β subunit isoform expressed in T-cell precursors in the thymus. Knockout of *Prkab1* caused earlier onset of lymphomas in both homozygous and heterozygous p53 knockouts, suggesting that β1 had a tumour suppressor role [147]. However, in both this model and the previous one, the knockouts of AMPK genes (*Prkaa1* or *Prkab1*) were global rather than T-cell specific, so it was not possible to conclude that these were cell-intrinsic effects on AMPK in the tumour progenitor cells themselves. Another mouse model used prostate -specific knockouts of both the tumour-suppressor gene *Pten* and *Prkab1*; although the additional knockout of AMPK-β1 did not affect prostate size, it did result in a higher proliferative index and pathological grade of tumour [148]. A drawback with this model was that the prostate gland also expresses AMPK-β2, which might have partially compensated for lack of β1 and might explain why the effects on tumourigenesis were relatively modest.

These various limitations were recently overcome in a mouse model of T cell acute lymphoblastic leukaemia/lymphoma (T-ALL), in which one or both of PTEN and AMPK-α1 were knocked out in a conditional manner in T-cells which (as already mentioned) do not express AMPK-α2 [149]. Knockout of *Prkaa1* alone did not cause the appearance of any lymphomas, but it accelerated the development of lymphomas induced by loss of the *Pten* gene. As in the model of B-cell lymphoma discussed above [146], this was associated with hyperactivation of mTORC1, and increased expression of HIF-1α and glycolytic enzymes, over and above that observed upon knockout of *Pten* only. These results show that the presence of AMPK-α1 protects, in a cell-intrinsic manner, against the development of T-ALL triggered by PTEN loss.

**Figure 9:**
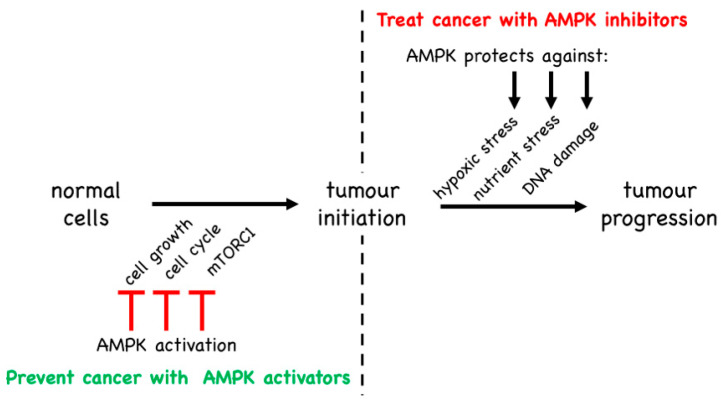
The central dichotomy in the functions of AMPK as a target in cancer therapy. LEFT: by inhibiting biosynthesis and thus cell growth, the cell cycle and thus cell proliferation, and mTORC1 and thus metabolic changes conducive to cell growth, AMPK activators such as phenformin can prevent or delay cancer initiation. RIGHT: once cancer has arisen, AMPK may switch to promoting survival of cancer cells, by protecting them against the hypoxic, nutrient/energy and DNA replication stresses to which they might otherwise become vulnerable.

A further question was addressed in this study: since the presence of AMPK-α1 in the tumour progenitor cells provided protection against T-ALL, would additional protection be provided by treatment with pharmacological activators of AMPK? These experiments were inspired in part by earlier findings that the use of metformin to treat Type 2 diabetes, compared with alternative treatments, is associated with a lower incidence of cancer in humans [150]. Although this association has been observed across many diabetic cohorts [151], it remained unclear whether: (i) it was mediated by AMPK activation by metformin; and (ii) it was a cell-intrinsic effect in the tumour progenitor cells themselves (for example, it might have been due to the ability of metformin to lower plasma glucose and/or insulin levels due to effects in other tissues or organs, such as the liver). The mouse model of T-ALL [149] provided an excellent opportunity to answer these questions. Somewhat disappointingly, oral treatment with metformin had no effect on the time of onset or the rate of appearance of T-ALL induced by PTEN loss, irrespective of the presence or absence of AMPK [149]. However, this could be ascribed to the demonstrated inability of the drug to enter developing T-cells in the thymus, most likely due to a lack of expression of members of the organic cation transporter family such as OCT1, which are required for metformin uptake [149]. It was already known that the more hydrophobic biguanide drug phenformin could readily enter cells lacking organic cation transporters [114], so phenformin was also tested. Intriguingly, phenformin treatment significantly delayed the time of onset and the rate of appearance of T-ALL induced by PTEN loss, but only when AMPK-α1 was present in the tumour progenitor cells. This demonstrated not only that the tumour suppressor effect of phenformin was AMPK-mediated but also, since all cells other than T-cells in this conditional knockout model would have been expressing normal levels of AMPK-α1, that this was a result of cell-intrinsic activation of AMPK in the tumour progenitor cells themselves. Notably, of the mice that expressed AMPK in T-cells treated with phenformin, four out of 14 had not developed any lymphomas by the termination of the experiment at 250 days of age, compared with only one out of 35 in the untreated group. Interestingly, similar results had been previously obtained with mice that were heterozygous for PTEN in a whole-body context, in which oral treatment with metformin, phenformin or A-769662 significantly delayed tumour formation in various organs or tissues [152]. Although these results show that AMPK-activating drugs can protect against the development of cancer, it is worth noting that in both cases the treatment was started before any tumours had become evident. If these findings do have any relevance to medical practice in humans, it might therefore be that AMPK activators could be used to prevent or delay cancer in individuals who were at high risk of its development because of other genetic factors, analogous to the mice with the T-cell-specific knockout of PTEN (Figure 9). Another point to note here is that phenformin was withdrawn for the treatment of Type 2 diabetes in 1978 because its use was associated with cases of lactic acidosis. This side-effect occurs with metformin as well as phenformin, although it is much less common with the former (≈three cases per 100,000 patient-years, as opposed to >60 with phenformin [153]). Nevertheless, even with phenformin, this complication (while life-threatening) is rare, and the risk may be more acceptable during prevention of cancer than for long-term treatment of diabetes.

Other evidence supporting the idea that AMPK is a tumour suppressor comes from studies of oncoproteins that are ubiquitin ligases and that appear to be involved in the cellular degradation of AMPK. For example, MAGE-A3/-A6 are two closely related members of the melanoma antigen family, which are normally only expressed in testis but become aberrantly re-expressed in some tumours (hence their designation as tumour antigens) [154]. They bind to the ubiquitin E3 ligase TRIM28, and a screen revealed AMPK-α1 to be a target for polyubiquitylation by this complex, resulting in its proteasomal degradation. Supporting this, knockdown of MAGE-A3/A6 or TRIM28 in tumour cells increased the expression of AMPK-α1 and triggered changes in metabolism and signalling that would be expected downstream of AMPK, including inhibition of mTORC1. Moreover, various human tumour cells that express MAGE-A3/-A6 have reduced levels of AMPK-α1 protein [154]. Another example is provided by the cancer-associated ubiquitin ligase, UBE2O [155]. As expected for an oncogene, knockout of the *Ube2o* gene in mice was found to attenuate tumourigenesis in models of both breast and prostate cancer. Interestingly, AMPK-α2 was identified as a UBE2O-interacting protein that is targeted for polyubiquitylation and consequent proteasomal degradation by the ubiquitin ligase. Consistent with this, expression of AMPK-α2 (but not -α1) was upregulated in *Ube2o* knockout mice. A human colon cancer cell line also grew less rapidly in mouse xenografts when *UBE2O* was knocked down using shRNA, and this was reversed by the simultaneous knockdown of AMPK-α2, but not -α1. Finally, the *UBE2O* gene in humans is located at a region of chromosome 17 that is amplified in many cancers. Using immunohistochemistry, there was a negative correlation between expression of UBE2O and AMPK-α2, but a positive correlation with S6 phosphorylation, indicating up-regulation of the mTORC1 pathway, in human breast cancers. Taken together, these results suggest that either AMPK-α1 or AMPK-α2 can act as tumour suppressors in human cancer, depending upon the cellular context.

### 5.2. Evidence from Mouse Models That AMPK Is a Tumour Promoter

As discussed in the previous section, some of the strongest evidence in favour of AMPK being a cell-intrinsic tumour suppressor came from a study of a mouse model of T-ALL [149]. Surprisingly, studies of the same cancer type using a different murine model came up with the opposite conclusion [156]. In the latter study, T-ALL tumour cells were generated in vitro by retroviral expression of an oncogenic NOTCH1 mutant in mouse haematopoietic stem cells that also carried a floxed *Prkaa1* (AMPK-α1) gene and a Cre recombinase gene driven by a tamoxifen-inducible promoter. The transformed cells were multiplied in irradiated mice and then injected into secondary irradiated recipient mice. After allowing the disease to become established for 10 days, these mice were then treated with tamoxifen to acutely delete AMPK-α1 in the T-ALL cells in vivo. In this model, knocking out AMPK reduced the recovery of T-ALL cells in spleen, lymph nodes and bone marrow, and enhanced survival of the mice [156]. Thus, the presence of AMPK-α1 was promoting the proliferation of T-ALL cells and making the disease more aggressive, suggesting that AMPK-α1 was acting as a tumour promoter. How can this be reconciled with the study described in Section 5.1, where AMPK-α1 appeared to be acting as a tumour suppressor in a different mouse model of T-ALL? The key difference between these studies is that, in the latter [149], AMPK had been knocked out, or treatment with AMPK-activating drugs initiated, prior to the development of disease, whereas in the other study [156], AMPK was present while the disease was developing and was only knocked out after it had become established. By inhibiting cell growth and proliferation, and by opposing the activation of pathways that promote those processes such as the mTORC1 pathway, it seems reasonable that AMPK would oppose the initiation of tumours, thus acting as a tumour suppressor. However, if (despite the best efforts of AMPK) tumours nevertheless did become established due to activating mutations in proto-oncogenes or loss-of-function mutations in other tumour suppressors, then AMPK may simply switch to become a tumour promoter instead (Figure 9). Note that the AMPK system most likely evolved as a stress response system that protected single-celled eukaryotes against energetic or nutritional stress. This ancestral, cell-intrinsic function remains relevant in cells of multicellular organisms today, irrespective of whether they are normal cells or tumour cells. Because their rapid growth often outstrips the ability of their blood supply to provide adequate nutrients and oxygen, tumour cells (and solid tumours in particular) will often be under energetic or nutritional stress. Paradoxically, by slowing cell growth and proliferation once it is activated by the stress, and by adjusting metabolism to respond to the stressful situation, the presence of AMPK may aid survival of the tumour cells and thus in the long term have a tumour-promoting effect, which would be good for the cancer cells but bad for the patient. We therefore propose that, while activators of AMPK may prove to be useful in prevention of cancer, most likely in individuals who have a high genetic risk of developing the disease, inhibitors of AMPK may instead be efficacious in the treatment of pre-existing cancers.

Another study suggesting that AMPK can promote tumour growth involved a mouse model of non-small cell lung cancer in which the tumours develop in situ at their site of origin, and in which both AMPK-α1 and -α2 were knocked out [157]. Mice harbouring Lox-STOP-Lox alleles of the *Kras^G12D^* oncogene and firefly luciferase were crossed with mice expressing floxed alleles of the genes encoding p53, LKB1, and AMPK-α1 plus -α2. Cre-recombinase was delivered to the lungs by nasal inhalation of lentiviral vectors, triggering recombination at twin *loxP* sites in a small subset of lung epithelial cells, so that expression of the *Kras^G12D^* oncogene and luciferase would be switched on, while different combinations of p53, LKB1, and AMPK-α1 plus -α2 would be simultaneously knocked out; growth of tumours could be monitored in vivo via bioluminescence from the expressed luciferase. Knockout of LKB1 enhanced growth of tumours expressing mutant K-Ras as reported previously [158] but, by contrast, knockout of both AMPK-α1 and -α2 was found to cause reductions in the size and number of lung tumours in *Kras^G12D^ Trp53^−/−^* mice. Overall, these results confirmed that LKB1 is a tumour suppressor in non-small cell lung cancer, while the presence of both AMPK-α1 or -α2 promoted tumour growth [157].

### 5.3. Evidence from Genetic Changes in AMPK Genes in Human Cancer

Although the most definitive evidence for the contrasting roles of AMPK in cancer has come from the study of mouse models, analysis of genetic changes that are associated with human cancers can provide revealing clues and circumstantial evidence. The cBioPortal database [159,160] provides a user-friendly platform to interrogate genetic changes occurring in human cancers for any particular gene, based on the numerous genomic studies of different cancer types that have been performed. Recent analyses of this database [1,140,161] revealed that, while the *PRKAA2* gene encoding AMPK-α2 is often mutated in human cancers, the *PRKAA1* gene encoding AMPK-α1 (also, interestingly, the *PRKAB2* gene encoding AMPK-β2) is often amplified instead. While not providing conclusive proof, these results suggest that, if anything, AMPK-α2 may act as a tumour suppressor in human cancers, while AMPK-α1 (and -β2) are more likely to represent tumour-promoting genes. In fact, some evidence for a similar isoform selectivity was obtained with studies of transformed mouse embryo fibroblasts (MEFs) performed several years ago [162]. Primary MEFs from wild type (WT), AMPK-α1 or AMPK-α2 knockout (KO) mice were transformed by expression of V12 mutant H-Ras in vitro; the α1-KO cells grew at a slow rate similar to that of the WT, while the α2-KO cells grew significantly more rapidly that the WT cells and, unlike the latter, also formed colonies in soft agar. Consistent with this, when grown as solid tumours in the flanks of nude mice, only the α2-KO cells grew rapidly; the WT cells grew much more slowly after a longer lag phase, while the α1-KO cells did not grow at all [162]. These findings are consistent with the view that, while AMPK-α2 is a tumour suppressor in that tumour growth is accelerated when it is lost, AMPK-α1 is a tumour promoter whose presence is required for the growth of cancer cells in vivo.

For reasons that remain unclear, mutations in the *PRKAA2* gene are particularly frequent in human skin cancer and melanoma, where, in several different studies, they occurred in 10–23% of cases, while amplification of *PRKAA1* is common in adenocarcinoma and squamous cell carcinoma of the lung, where it has been found to occur in 8–11% of cases [140]. Although the proportion of the cancer mutations in *PRKAA2* that cause loss-of-function is not yet clear, the occurrence of frequent mutations suggests that, if anything, AMPK-α2 is acting as a tumour suppressor. By contrast, the fact that the genes encoding AMPK-α1 and -β2 are frequently amplified suggests that these are tumour promoters where gene amplification is being selected for because it aids tumour cell growth. One caveat with this is that gene amplifications in cancer do not usually just affect single genes, but instead whole segments of chromosomes containing multiple genes. It therefore remains possible that *PRKAA1* (and/or *PRKAB2*) are located in the genome near to another oncogene whose amplification is being selected for in the tumour cells, with the AMPK genes merely being innocent passengers. Arguing against this in the case of *PRKAA1,* however, is analysis of simultaneous genetic changes in known oncogenes and tumour suppressors that occur within the same cases of cancer. For example, in 230 cases of lung adenocarcinoma studied by The Cancer Genome Atlas (TCGA) network [163], the *PRKAA1* gene was amplified in 22 (10%), while the *STK11* gene (encoding the upstream kinase LKB1) was subject to either deletions, or mutations expected to cause loss-of-function, in 43 (19%). However, these changes never occurred together in the same case. The probability (*p*) that this would occur by random chance if the two genes were behaving independently was <0.005 [1]. These results suggest that the increased activity of AMPK-α1 due to gene amplification had been the subject of positive selection, because if the kinase activity of AMPK was irrelevant to the function of the cancer cells, there would have been no necessity to maintain LKB1 function.

Two other genes subject to particularly frequent mutations in lung adenocarcinomas are *KRAS,* encoding the K-Ras variant of the small G protein Ras (36% of cases), and *TP53,* encoding the tumour suppressor and transcription factor p53 (47% of cases). Interestingly, in the TCGA study [163] there was a significant mutual exclusion between amplification of *PRKAA1* and mutations in *KRAS* (*p* < 0.005), but a significant co-occurrence with mutations in *TP53* (*p* < 0.001) [1]. In the 22 cases of lung adenocarcinoma with amplification of *PRKAA1*, only 3 were not also associated with loss-of-function mutations in *TP53* [140]. Thus, amplification of AMPK-α1 (and normal function of LKB1) seems to be selected for in tumours driven by the loss of p53 function—why should this be? The classical role of p53 is to become stabilized or activated in response to DNA damage, and to cause a G1 cell cycle arrest, which it achieves by inducing transcription of genes such as *CDKN1A* encoding the G1 cyclin-dependent kinase inhibitor p21^CIP1^. This allows time either for the DNA damage to be repaired, or for further proliferation of the aberrant cell to be prevented by induction of senescence or apoptosis [164]. AMPK, and specifically the α1 isoform that is amplified in some cancers, is selectively activated in the cell nucleus by genotoxic treatments, providing protection against cell death induced by such treatments, at least in part by up-regulating expression of p21^CIP1^ [7]. Thus, amplification of the *PRKAA1* gene may be being selected for in *TP53*-mutant tumours because it can compensate for the lack of p53 and protect the tumour cells against genotoxic stresses that might otherwise kill them. If this hypothesis is correct, then AMPK inhibitors, especially if α1-selective, might be a particularly effective adjunct to cytotoxic treatments of p53-mutant tumours.

### 5.4. Role of AMPK in Cancer Stem Cells

Recently, interest has been growing in the role of cancer stem cells (CSCs) in the initiation, progression, resistance to drug therapy and recurrence of tumours. AMPK has been suggested either to hinder or promote the development of CSCs, although the evidence for the latter view is more limited. Moreover, AMPK activation has been reported to restore the sensitivity of cancer cells to chemotherapeutics.

A significant body of evidence exists suggesting a role for metformin, a known activator of AMPK (Section 4.2), in reduced survival or inhibition of growth of cancer stem cells, as well as inhibition of their differentiation potential and expression of stemness markers [165,166,167,168,169,170,171]. Many of these studies used metformin at concentrations of 1–10 mM or even higher, which is well above the peak plasma concentrations of metformin when given at normal doses in humans. However, Struhl’s laboratory [171] reported effects of metformin on CSCs derived from breast cancers at the more pharmacologically relevant concentration of 100 µM. Interestingly, at this dose metformin synergized with a variety of chemotherapeutics in a range of cancer cell lines to block tumour growth and prevent relapse [172]. Metformin at 30–100 µM also induced radiosensitization of pancreatic cancer cells in an AMPK-dependent manner [173]. In addition, both metformin and A-769662 (the latter a direct activator of AMPK that acts in a manner different from metformin by binding at the ADaM site, Section 4.3), were reported to impede the generation of induced pluripotent stem cells (iPSC) from fibroblasts [174].

It is now clear that metformin has some metabolic effects that are not mediated by AMPK (e.g., [91,92]), so it was important to demonstrate that the effects of metformin on CSCs were dependent upon AMPK. Supporting this, AMPK knockdown using siRNA reduced the differentiation-promoting effects of metformin on stem-like glioma-initiating cells [175]. In another study [176], a CSC-like phenotype was induced in hepatocellular carcinoma cells by selecting for a cell population resistant to the ant-cancer drug sorafenib; the resulting cells had impaired AMPK signalling and increased expression of stem cell markers. However, both stemness and the induced drug resistance could be reduced or reversed either by exogenous expression of AMPK, or by its activation using A-769662. Conversely, knockdown of AMPK using siRNA in the original cell lines was sufficient to induce expression of stemness markers and resistance to drug treatment [176]. 

A number of mechanisms have been suggested to explain regulation of stemness by AMPK, primarily related to the modulation of expression of pluripotency-related transcription factors. Thus, AMPK inhibits activation of STAT3, a transcription factor that activates expression of the iPSC markers NANOG, OCT4 and SOX2 [177]. AMPK was also reported to induce the degradation of NANOG by inhibiting its BRAF-dependent phosphorylation, resulting in increased interaction with an E3 ubiquitin ligase adaptor protein, SPOP, and subsequent ubiquitin-mediated degradation [178].

If AMPK does indeed suppress CSCs, we find ourselves in the paradoxical situation where inhibition of AMPK might inhibit the growth of the bulk of the tumour, while potentially also taking the brakes off the proliferation of tumour-initiating cancer stem cells. In such a situation, AMPK activators (rather than inhibitors) might be useful to prevent metastasis after chemotherapy or surgery. However, as always with AMPK, the cellular context appears to be critical. In contrast to the studies cited above, Saito et al. [179] have demonstrated that, under conditions of severe metabolic stress (a combination of dietary restriction and the hypoxic environment of the bone marrow), AMPK deletion significantly reduced the population of leukaemia-initiating cells in the bone marrow, but not in the relatively more benign environment of the spleen [179].

## 6. Conclusions

Our understanding of the role of AMPK in cancer is continually evolving, which is perhaps not surprising given the complex, multifaceted functions of this kinase and the heterogeneous nature of the disease. Much has been done in the last few years to begin to refine our models of the actions of AMPK in cancer, and exciting times undoubtedly lie ahead. We await with great interest studies examining the potential of AMPK inhibitors to treat established cancers, although the lack of truly specific inhibitors is a current impediment—we therefore also hope to see much more selective inhibitors emerging from the drug development pipelines. 

The disparity in the function of AMPK in different cancer models suggests that it might well be a target for the developing field of personalised medicine. Clearly, AMPK inhibitors would be expected to be particularly effective in metabolically compromised tumours that have amplification of the *PRKAA1* gene, as is seen in a significant proportion of lung cancers. Given the high incidence of mutations of AMPK-α2 in cancer, especially in skin cancer and melanoma, the ideal inhibitor would also be selective, if not completely specific, for the α1 isoform. However, even non-isoform-selective AMPK inhibitors, by rendering cells more sensitive to DNA damage, may have potential to be effective adjuncts when administered with conventional cytotoxic treatments.

## Abbreviations

ACC	Acetyl-CoA carboxylase
ADaM	Allosteric drug and metabolite (binding site on AMPK heterotrimer)
AICA	5-aminoimidazole-4-carboxamide
α-AID	Autoinhibitory domain of the AMPK-α subunit
AMPK	AMP-activated protein kinase
ARK	AMPK-related kinase
ASA	Acetyl salicylic acid
*ATIC*	Human gene encoding both AICAR transformylase and IMP cyclohydrolase
ATM	Serine-protein kinase ATM (Ataxia telangiectasia mutated)
CaMKI	Calmodulin-dependent kinase-I
CaMKK2	Ca^2+^/calmodulin-dependent kinase kinase-2 (-β)
β-CBM	Carbohydrate-binding module of the AMPK-β subunit
CBS	Cystathionine β-synthase (refers to CBS domain or CBS repeat)
CBS1	CBS repeat 1; refers to an adenine nucleotide-binding site on the AMPK-γ subunit
CBS3	CBS repeat 3; refers to an adenine nucleotide-binding site on the AMPK-γ subunit
CBS4	CBS repeat 4; refers to an adenine nucleotide-binding site on the AMPK-γ subunit
C-lobe	C-terminal lobe of a kinase domain
CSCs	Cancer stem cells
α-CTD	C-terminal domain of the AMPK-α subunit
β-CTD	C-terminal domain of the AMPK-β subunit
EXO1	Exonuclease 1
FBP	Fructose-1,6-bisphosphate
GHSR1	Growth hormone secretagogue receptor-1
IGF-1	Insulin-like growth factor-1
IP3	Inositol-1,4,5-triphosphate
iPSCs	Inducible pluripotent stem cells
α-KD	Kinase domain of the AMPK-α subunit
KO	Knockout
LCFA	Long chain fatty acid
α-linker	Linker region of the AMPK-α subunit
LKB1	Liver kinase B-1
MAGE	Melanoma antigen gene (a gene family)
MEFs	Mouse embryonic fibroblasts
MO25	Mouse protein 25
mTORC1	mammalian (or mechanistic) target-of-rapamycin complex-1
NES	Nuclear exclusion sequence
N-lobe	N-terminal lobe of a kinase domain
NLS	Nuclear localization sequence
NOTCH1	Neurogenic locus notch homolog protein 1
OCT1	Organic cation transporter 1
*PPP1R3C*	Human gene encoding a glycogen-targetting regulatory subunit of protein phosphatase-1
*PRKAA1*	Human gene encoding AMPK-α1
*PRKAA2*	Human gene encoding AMPK-α2
*PRKAB1*	Human gene encoding AMPK-β1
*PRKAG1*	Human gene encoding AMPK-γ1
*PRKAG2*	Human gene encoding AMPK-γ2
*PRKAG3*	Human gene encoding AMPK-γ3
PTEN	Phosphatase and tensin homolog protein
shRNA	Short hairpin RNA
siRNA	Small interfering RNA
STRAD	Ste20-related adaptor
*STK11*	Human gene encoding LKB1
ST loop	Serine/threonine-rich regulatory loop near the C-terminus of the AMPK-α subunit
T-ALL	T cell acute lymphoblastic leukameia/lymphoma
TCGA	The Cancer Genome Atlas
TRIM-28	Tripartite motif containing 28 (ubiquitin E3 ligase)
UBE2O	E2/E3 hybrid ubiquitin-protein ligase
VEGF	Vascular endothelial cell growth factor
ZMP	5-aminoimidazole-4-carboxamide ribonucleoside monophosphate
